# Multiplex Lithographic SERS Aptasensor for Detection of Several Respiratory Viruses in One Pot

**DOI:** 10.3390/ijms24098081

**Published:** 2023-04-29

**Authors:** Vladimir Kukushkin, Oganes Ambartsumyan, Alexei Subekin, Anna Astrakhantseva, Vladimir Gushchin, Alexandra Nikonova, Anastasia Dorofeeva, Vitaly Zverev, Anna Keshek, Nadezda Meshcheryakova, Olga Zaborova, Alexandra Gambaryan, Elena Zavyalova

**Affiliations:** 1 Osipyan Institute of Solid State Physics, Russian Academy of Science, 142432 Chernogolovka, Russia; anna.sergeyenkova@phystech.edu; 2 Moscow Institute of Physics and Technology, 141701 Dolgoprudny, Russia; ogan-mail@mail.ru (O.A.); alexey.subekin@gmail.com (A.S.); 3 N. F. Gamaleya Federal Research Center for Epidemiology & Microbiology, 123098 Moscow, Russia; wowaniada@gmail.com; 4 Mechnikov Research Institute of Vaccines and Sera, 105064 Moscow, Russia; aa.nikonova@nrcii.ru (A.N.); d0r0feeva@yandex.ru (A.D.); vitalyzverev@outlook.com (V.Z.); 5 Chemistry Department of Lomonosov, Moscow State University, 119991 Moscow, Russia; akeshek@list.ru (A.K.); n.f.mescheryakova@gmail.com (N.M.); colloid.chem.msu@gmail.com (O.Z.); 6 Chumakov Federal Scientific Center for Research, Development of Immune and Biological Products RAS, 108819 Moscow, Russia; al.gambaryan@gmail.com

**Keywords:** adenovirus, aptamer, aptasensor, influenza virus, sensor, SARS-CoV-2, SERS, respiratory syncytial virus, virus

## Abstract

Rapid and reliable techniques for virus identification are required in light of recurring epidemics and pandemics throughout the world. Several techniques have been distributed for testing the flow of patients. Polymerase chain reaction with reverse transcription is a reliable and sensitive, though not rapid, tool. The antibody-based strip is a rapid, though not reliable, and sensitive tool. A set of alternative tools is being developed to meet all the needs of the customer. Surface-enhanced Raman spectroscopy (SERS) provides the possibility of single molecule detection taking several minutes. Here, a multiplex lithographic SERS aptasensor was developed aiming at the detection of several respiratory viruses in one pot within 17 min. The four labeled aptamers were anchored onto the metal surface of four SERS zones; the caught viruses affect the SERS signals of the labels, providing changes in the analytical signals. The sensor was able to decode mixes of SARS-CoV-2 (severe acute respiratory syndrome coronavirus two), influenza A virus, respiratory syncytial virus, and adenovirus within a single experiment through a one-stage recognition process.

## 1. Introduction

The COronaVIrus Disease 2019 (COVID-19) pandemic caused the deaths of more than 6 million people worldwide [1]. The new threat increased interest in the development of new rapid methods for respiratory viral infection diagnostics. One of the main problems is the differential diagnostics between respiratory viruses such as severe acute respiratory syndrome coronavirus two (SARS-CoV-2), influenza, adenovirus, and respiratory syncytial virus which can cause similar clinical symptoms such as fever, chills, cough, shortness of breath, fatigue, sore throat, and headache [2,3]. In this regard, it is important to develop new multiplex rapid diagnostic techniques that allow the detection of pathogens simultaneously and, therefore, provide rapid and early diagnosis of respiratory viruses, preventing infections from spreading and reducing the time of analysis and its cost.

The development and improvement of multiplex analysis systems based on surface-enhanced Raman spectroscopy (SERS) is an urgent trend. The SERS technique has a number of important advantages:

− high sensitivity (up to 10^−12^–10^−16^ M of the substance in the sample);
− the peaks of the SERS spectra have a narrow spectral band compared to fluorescence, which simplifies spectral separation in complex media;
− the compatibility with antibodies, aptamers, and DNA probes allows specific detection of various proteins, genomes, viruses, cells, etc.

To date, the possibility of multiplex detection using SERS has been repeatedly demonstrated.

Wang et al. proposed a SERS lateral flow immunoassay (SERS-based LFIA) strip for the influenza A (H1N1) virus and human adenovirus (ADV) detection by using magnetic SERS nanotags. Magnetic tags, conjugated with dual-layer Raman dye molecules and target virus-capture antibodies, designed for specific recognition and magnetic enrichment of target viruses in the solution and SERS detection of the viruses on the strip. The limits of detection for H1N1 and ADV were 50 and 10 pfu/mL, respectively, and the SERS strip can be used for biological samples without any sample preparation [4].

Another example of a SERS-based multiplex sensor with dried reagents was shown by Sebba et al. Capture antibodies for the Ebola, Lassa, and malaria biomarkers are conjugated to specific SERS nanotags and magnetic nanoparticles, which are stored dried in a test tube. A whole blood sample and a lysis buffer were added to rehydrate the dried reagents. After that, the magnetic microparticles–antigen–SERS nanotag complexes were separated with an external magnet, and the SERS signal was measured. The limit of detection of Ebola and Lassa viruses was 10^5^ copies/mL [5].

One more SERS biosensor is a lateral flow sandwich-type microarray, which is a 2×3 microarray on a lateral flow strip with the ability to simultaneously detect 11 common respiratory pathogens. A lateral flow microarray was created by immobilizing captured nucleic acids, complementary to pathogenic ones, on a nitrocellulose membrane surface. The target-pathogen nucleic acids from the samples were bound by the complementary nucleic acids fixed to the surface of the SERS nanotags. The limits of detection (LoD) for influenza A, parainfluenza one, parainfluenza three, respiratory syncytial virus, *Coxiella burnetii*, *Legionella pneumophila*, influenza B, parainfluenza two, adenovirus, *Chlamydophila pneumoniae*, and *Mycoplasma pneumoniae* were estimated to be in the range of 0.030–0.041 pM [6].

Liu et al. presented a multichannel surface-enhanced Raman scattering-based lateral-flow immunoassay using magnetic SERS tags for the simultaneous detection of influenza A virus (H1N1), SARS-CoV-2, and respiratory syncytial virus (RSV) in biological samples. In that scheme, magnetic SERS tags form a sandwich-like structure on the test lines together with target viruses and antibodies. The limit of detection of the assay is 85 copies/mL, 8 pg/mL, and 8 pg/mL for H1N1, SARS-CoV-2, and RSV, respectively [7].

Many promising studies are ongoing through over the world. The ideal test is to be cheap, easy to implement, has low LoDs, scalable production technology, and a detection principle that is transferrable to other analytes. The final choice is unobvious today. Therefore, the new combinations of analytical techniques, recognition elements, and technical implementation are of high interest.

Here, an alternative setup for a multiplex sensor is provided. The previous work with an excellent limit of detection for the SARS-CoV-2 virus (100 viral particles/mL) used a single recognition element, SARS-CoV-2 binding aptamer labeled with Cyanine-3 dye, for a one-stage procedure. The virus binding changed the distance between the dye and the lithographic metal surface providing the change in the SERS signal from the dye. The sensor detected as low as two viral particles per assay, reliably enabling the detection of a single pathogen in the probe [8]. This simple one-stage approach has been used to create multiplex sensors for the simultaneous detection of four respiratory viruses.

## 2. Results and Discussion

### 2.1. Specificity of the Aptamers

The idea of a multiplex sensor presupposes no cross specificity between the recognizing elements. Four aptamers targeting four viruses with minimal cross specificity were chosen. An RHA0385 aptamer was selected to bind the hemagglutinin of the influenza A virus [9]; it is known to bind influenza A viruses of the H5N1 and H7N1 subtypes [10,11]. H8 aptamer was selected for the respiratory syncytial virus (RSV) A2 subtype [12]. RBD-1C aptamer was selected for the receptor binding domain of the S protein of SARS-CoV-2 [13]; it was shown to bind the SARS-CoV-2 virions [14]. ADV-20 aptamer was selected for the adenovirus subtype two [15].

**Figure 1:**
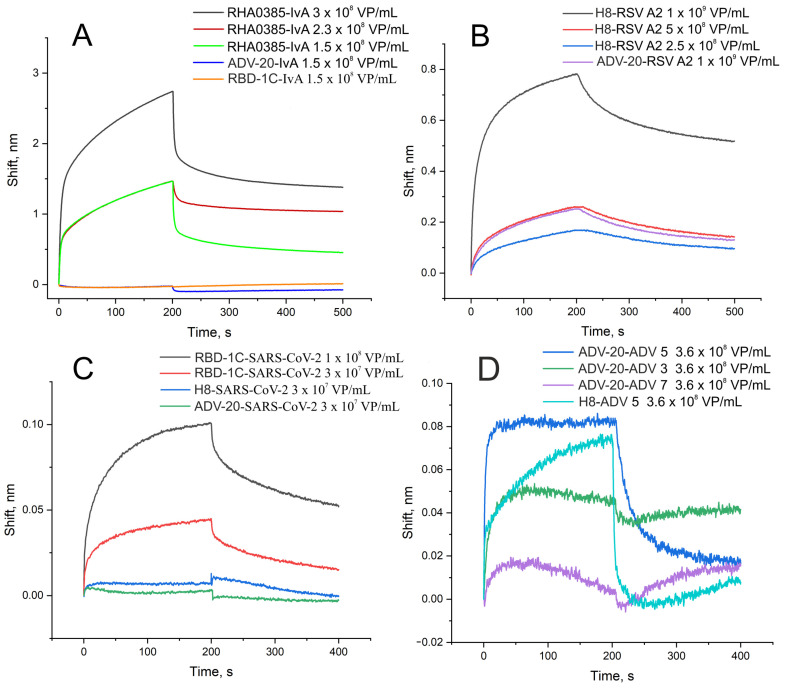
Interaction between aptamers and viruses assessed by biolayer interferometry. (**A**) Binding of specific aptamer (RHA0385) and nonspecific aptamers to influenza A virus of H7N1 subtype. (**B**) Binding of specific aptamer (H8) and nonspecific aptamer to RSV. (**C**) Binding of specific aptamer (RBD-1C) and nonspecific aptamers to SARS-CoV-2. (**D**) Binding of specific aptamer (ADV-20) and nonspecific aptamer to adenoviruses type 3, 5, and 7. The association stage corresponds to 0–200 s; the dissociation stage corresponds to 200–500 s.

Biolayer interferometry showed that the binding of the specific aptamer to the virus provides either a higher signal or slower dissociation stage compared to a nonspecific aptamer with the same virus (Figure 1). The calculated dissociation constants are provided in Table 1. The typical dissociation constant was about 20 fM. The off-target complexes had dissociation constants at least 10 times higher compared to complexes of aptamers with target viruses. ADV-20 aptamer was shown to have a high affinity to adenovirus subtypes three and five additionally to the adenovirus subtype two that was reported originally.

### 2.2. Setup of the Aptasensor

**Figure 2:**
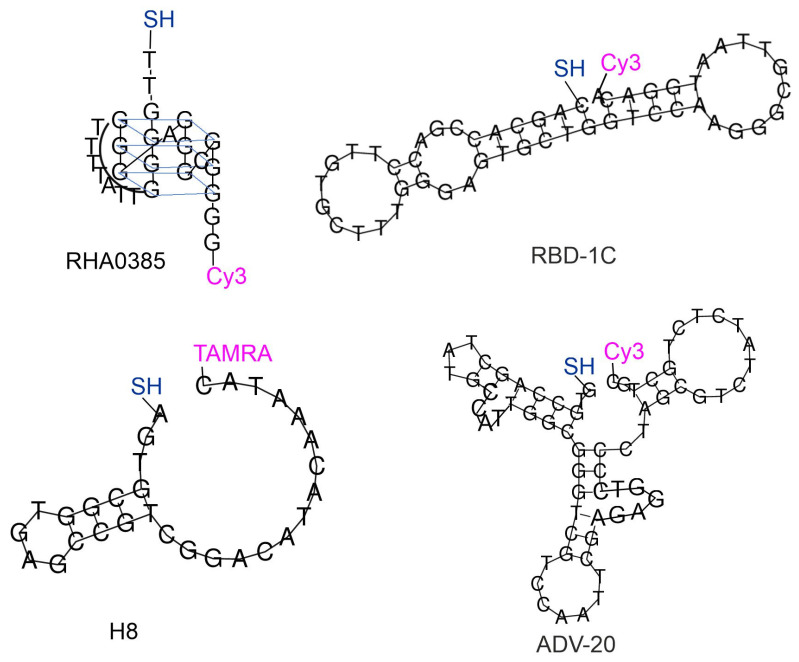
Four aptamers with modifications compatible with SERS-based sensors. The secondary structures of ADV-20, H8, and RBD-1C aptamers were built using RNAfold [16]. The structure of RHA0385 was built based on previous studies [10,17].

These four aptamers were synthesized with two modifications in each case. The thiol group was introduced at the 5′-end of the aptamers to immobilize the aptamer on the metal surface. Resonant Raman compounds, i.e., cyanine three (Cy3) or tetramethylrodamine (TAMRA) fluorescent dyes, were introduced at the 5′ ends of the aptamers (Figure 2). The same approach was used in previous work for the RBD-1C aptamer [8]. The ratio of surface-enhanced Raman signal to fluorescence intensity was decreased in the presence of the virus, providing an excellent limit of detection of SARS-CoV-2 of 100 copies/mL. Here we extended this approach to three other aptamers.

The analytical signal is provided by two possible scenarios. The first one includes the interaction of the labeled end of the aptamer with the target shielding the dye and removing it from the metal surface. This scenario was found for the complexes RBD-1C with SARS-CoV-2, RHA0385 with the influenza A virus, and H8 with RSV (See the next subsection for details). This mechanism of decrease in the analytical signal was supported with molecular modeling of the complex RBD of S protein of SARS-CoV-2 with RBD-1C, where the 3′ end of the aptamer is located near the protein surface [13]. The second scenario includes a conformational change of the oligonucleotide which pulled together the 5′ and 3′ ends of the aptamer. The analytical signal increases as the 5′ end is bound to the metal surface. This scenario was found for the complex of ADV-20 with adenovirus type five (See the next subsection for details).

**Figure 3:**
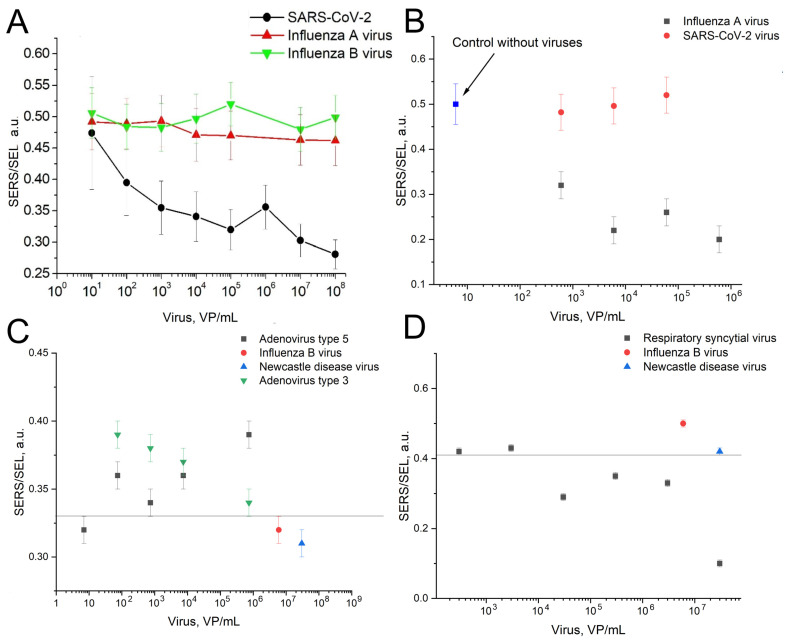
The dependences of the SERS to SEL ratio on the concentrations of the target viruses compared to the off-target viruses. (**A**)—RBD-1C aptamer to SARS-CoV-2 compared to influenza A and B viruses. Reproduced with permission from Kukushkin et al., Nanomaterials; published by MDPI, 2022 [8]. (**B**)—RHA0385 aptamer with influenza A virus compared to SARS-CoV-2. (**C**)—ADV-20 with adenoviruses type 3 and 5 compared to influenza B virus and Newcastle disease virus. The line indicates that the SERS/SEL value of 0.33 can be used to discriminate the virus-positive samples from the virus-negative samples. (**D**)—H8 aptamer with respiratory syncytial virus compared to influenza B virus and Newcastle disease virus. The line indicates that the SERS/SEL value of 0.41 can be used to discriminate the virus-positive samples from the virus-negative samples.

The overall setup is similar to the previously published one [8]. The aptamers were immobilized on the lithographic metal surface, whereas viruses were captured from liquid samples. The specificity and LoDs of the sensors were using the same lithographic substrates as in previous work [8]. The ratio of SERS to SEL (surface-enhanced luminescence) was changed in the presence of the target viruses and was not changed in the presence of the off-target viruses (Figure 3). The sensitivity of the sensors was estimated following the last virus dilution with statistically significant differences from the sample without the virus. These concentrations were 100 VP/mL for SARS-CoV-2, 600 VP/mL for the influenza A virus, 70 VP/mL of adenoviruses type three and five, and 3 × 10^4^ VP/mL for RSV. Figure 3C,D were supplemented with lines that separate the SERS/SEL values of the virus-positive samples from the virus-negative samples. The reference values were used for further analysis in the multiplex analysis.

### 2.3. Multiplex Sensor

The morphology of the SERS substrate was chosen based on the resonances of the combined metal–dielectric structure observed near the wavelength of the laser radiation used with a wavelength of 532 nm [18]. By using nanostructured SERS substrates and working with a liquid sample covered with a cover glass, it was possible to achieve very high reproducibility of the signal from the studied SERS tags. The relative standard deviation was about 3%.

In this work, a SERS substrate with four active zones was created. Each of them is a multilayer system based on an antenna array that provides the transformation of external electromagnetic waves into localized modes and surface plasmon polaritons at the interface between metal and dielectric. Such plasmon waves, unlike localized plasmons in single metal nanoparticles, make it possible to create sensors with predictable optical properties and achieve high enhancement factors at a selected photoexcitation wavelength.

The developed substrates have several advantages: they have a surface-reproducible enhancement factor (unlike nonlithographic substrates), provide a long-range mechanism for amplifying the Raman scattering signal (unlike colloidal SERS solutions), and suppress the luminescence signal from the dyes used (unlike flat nanostructured substrates).

**Figure 4:**
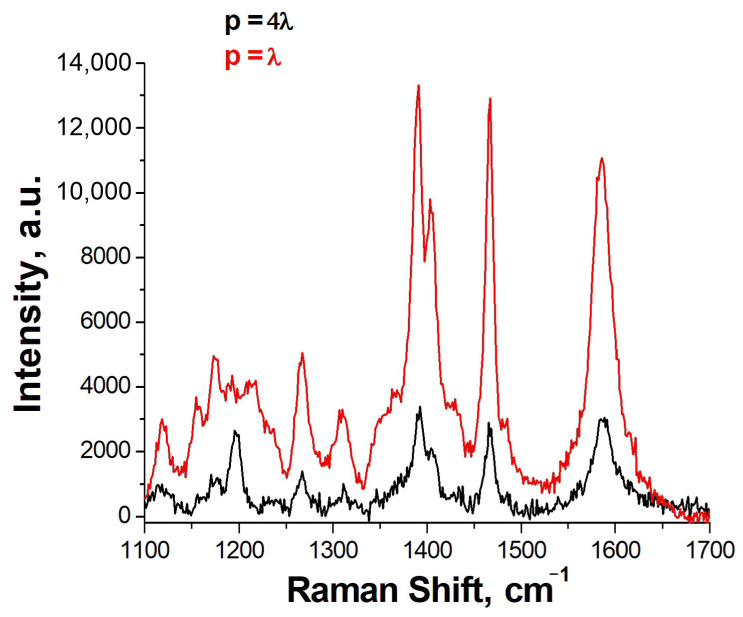
The SERS spectra of SH-RBD-1C-Cy3 on the previous lithographic substrates with the grating period (p) of the columns equal to 4λ and new lithographic substrates with p = λ.

Previously, we developed the aptasensor based on a lithographic SERS substrate with a grating period of SiO_2_ dielectric columns equal to 4λ, where λ is the wavelength of the exciting radiation (532 nm) [8]. In this work, we decided to make a multiplex sensor based on a periodic structure in which the main resonance is realized with a period equal to λ [19]. This approach allows the usage of the principal fundamental resonance that increases the apparent Q factor several times. The comparison of spectra of SH-RBD-1C-Cy3 is provided in Figure 4 where the SERS intensity is nearly four times higher for the new substrates compared to the previous design.

**Figure 5:**
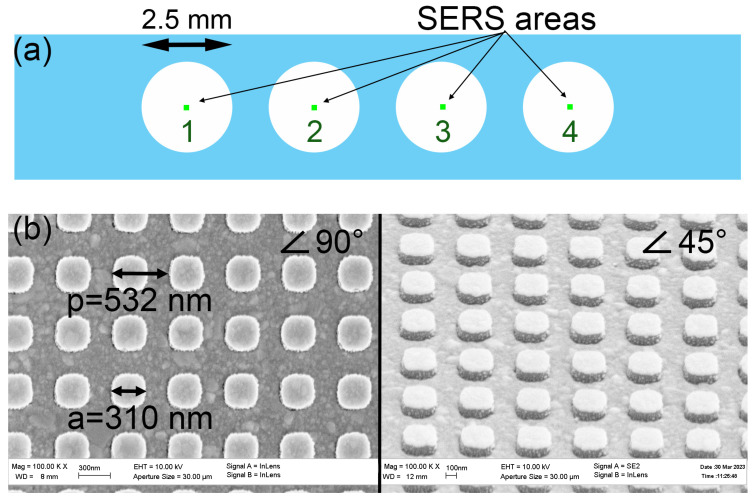
The scheme of the location of SERS zones inside the multisensory (**a**). Scanning electron microscope images of the lithographic SERS structures at an angle of 90 and 45 degrees (**b**).

The lithographic sensor was designed to have four zones (spots) (Figure 5). Each spot was modified with an individual aptamer; then, the whole sensor was incubated with a mixture of respiratory viruses (Table 2). The virus concentrations were chosen according to the typical concentration of SARS-CoV-2 in the samples derived from the nasal swabs that were in the range from 2 × 10^4^ to 1 × 10^7^ [20]. The viruses were diluted in a matrix solution of a medium from the noninfected cells imitating diluted biological samples acquired from the swab.

**Figure 6:**
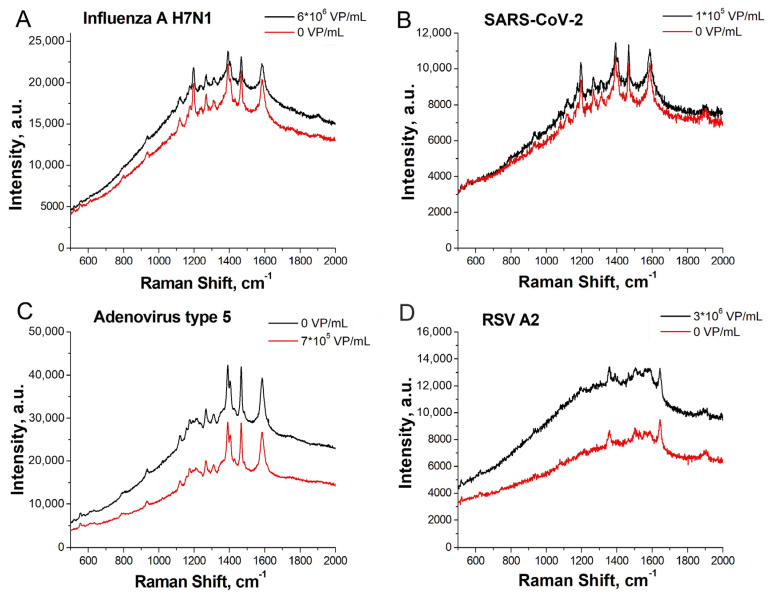
SERS spectra acquired with a multiplex lithographic SERS aptasensor. Comparison of sensors #1 and 2 from Table 2. Detection of influenza A virus H7N1 in spot b (**A**); SARS-CoV-2 in spot c (**B**); adenovirus type 5 in spot a (**C**); and RSV A2 in spot d (**D**).

**Figure 7:**
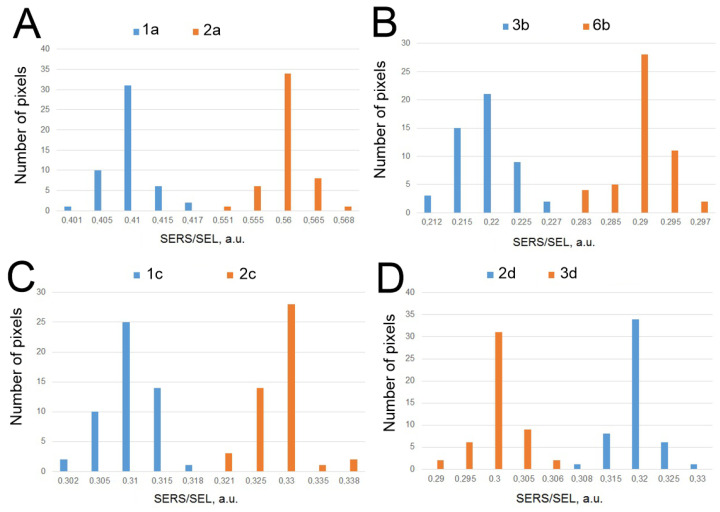
The differences between the virus-positive and virus-negative samples with the closest SERS/SEL ratio for spots modified with ADV-20 (**A**), RHA0385 (**B**), RBD-1C (**C**), and H8 (**D**) aptamers. The *p*-values were 0.99, 0.99, 0.99, and 0.95, correspondingly.

The SERS to SEL ratio was used as the main parameter for evaluation due to its reproducibility from sample to sample (Table 2, Figure 6). This parameter was decreased in the presence of the target virus in the cases of RBD-1C with SARS-CoV-2, RHA0385 with influenza A virus, and H8 with RSV. The analytical signal increased when the complex ADV-20 with adenovirus type five was assembled (See the previous subsection for the explanation of this result). The boundary values of the SERS to SEL ratio were estimated as the highest/lowest values in blank samples without the target virus derived from three independent samples. The differences between a virus-positive and a virus-negative sample were statistically significant with typical *p* values of about 0.99. The differences between the closest SERS/SEL ratio for the virus-positive and the virus-negative spots are shown in Figure 7.

The overall experiment shows a minimal if any cross-specificity for the aptasensors. In the current version, this sensor provides qualitative estimation only. The semiquantitative estimation is principally possible as demonstrated in the previous lithographic setup [8]. Further optimization of the aptamers could provide this significant property of the test system.

### 2.4. Comparison with Other Test Systems

The comparison of analytical performance in the multiplex detection of viral particles using aptamer-based SERS sensors, antibody-based SERS sensors, DNA probe-based SERS, SERS sensors without recognition elements, and conventional diagnostic assays are shown in Table 3. The table clearly indicates that some the SERS-based sensors are rapid tests (analysis time is below 20 min); whereas RT PCR (reverse transcription polymerase chain reaction), and RT LAMP (reverse transcription loop-mediated isothermal amplification) consume at least 1 h for the overall procedure. RT PCR has a low LoD that is not easily achieved by rapid tests. It is interesting to combine low LoD from the PCR technique with the simplicity and analysis time of the rapid tests. SERS-based tests can solve this issue.

SERS spectroscopy has many advantages over similar methods in determining various biological agents, especially in their multiplex detection. In this article, the simultaneous specific detection of four targets was successfully demonstrated, including RSV, adenovirus, influenza virus, and coronavirus. The results show the applicability of this method, not only for the rapid detection of a specific pathogen or group of familiar pathogens but also for the specific diagnosis of a set of various pathogens in a one-pot manner in a single experiment. The overall time of an analysis from sample preparation to result in acquirement was 17 min, only placing the sensor among the rapid tests. The LoD of the SERS aptasensor was of the same range as the LoD of the RT PCR placing the sensor among the sensitive test systems. The principal approach used in this multiplex sensor could be used for the creation of a chip with dozens of targets. Nucleic acid aptamers are unique recognizing elements for this purpose as they can provide analytical signals resulting from the complex formation with the target without any additional steps for signal production. DNA aptamers are stable during storage (nearly 1 day at rt, 1 week at +4 °C, and several months at −20 °C) and can be refolded easily using a short heating and cooling procedure. This feature highlights benefit of aptamers in comparison to proteins such as antibodies for practical implementation. The simplicity of the one-step process opens up broad prospects for the application of the aptamer-based multiplex analysis with SERS detection in various fields of practical implementation, including point-of-care applications for personalized diagnostics.

## 3. Materials and Methods

### 3.1. Reagents

Inorganic salts (AppliChem GmbH, Darmstadt, Germany and Sigma-Aldrich, St. Louis, MO, USA) and phosphate-buffered saline (PBS) tablets (Ecoservice, Saint Petersburg, Russia) were used. The DNA oligonucleotides were synthesized by Synthol (Moscow, Russia). The sequences are provided in Table 4.

To clean the substrate surface, extra-pure acetone and isopropyl alcohol (AO Reachem, Moscow, Russia) were used. MIBK (methyl isobutyl ketone, CAS Number: 108-10-1, Sigma-Aldrich, St. Louis, MO, USA) served as a developer for the PMMA electron beam lithography resist.

Four metals for evaporation, Cr, Ag, Au (GIRMET Ltd., Moscow, Russia), and Al (RD Mathis Company, Signal Hill, CA, USA) were used. In lithography processes, AZ5214E photoresist (MicroChemicals, Ulm, Germany) and AZ 726 MIF Developer (Merck, Darmstadt, Germany) were utilized.

### 3.2. Lithographic SERS-Substrate

SERS multiplex sensors were Si substrates with a 300 nm thick SiO_2_ top layer and polyester sticker. Four numbered active SERS areas with a size of 200 × 200 µm^2^ are located on the samples with dimensions of 20 × 4 mm^2^ (Figure 5A). All SERS areas were identical and represented dielectric pillars with a period of 532 nm and a height of 130 nm covered with a thick silver layer. Figure 5B depicts SEM (scanning electron microscope) images of the squared pillars. The polyester sticker had four holes, which opened the SERS areas (Figure 5A). Thus, SERS active areas were not covered by the sticker (located in the centers of holes) and were divided from each other by the walls of the polyester sticker with a thickness of 100 µm.

The substrates were fabricated as follows. Active regions were produced on a thermally oxidized silicon substrate using electron-beam lithography on a base of SEM (JSM-7001F JEOL, Tokyo, Japan) with an additional pattern generator (XENOS, Kusterdingen, Germany), resistive evaporation (NANO 38 system, Kurt J. Lesker Company, Jefferson Hills, PA, USA) of aluminum mask for etching, anisotropic reactive-ion etching of SiO_2_ in the mixture of gases SF6 and Ar (Oxford Plasmalab System 100, Oxford Instruments, Bristol, UK), and resistive evaporation of the silver layer (NANO 38 system). First of all, the Si/SiO_2_ substrates were cleaned with acetone and isopropyl alcohol in an ultrasonic bath (SW 1 H, Sonoswiss AG, Ramsen, Switzerland). Next, two layers of E-beam resist PMMA (ALLRESIST GmbH, Strausberg, Germany) were applied using a spin coater (Delta 6RC, SUSS MicroTec SE, Germany) with a spin rate of 6000 rpm. The first resist was AR-P 642.04, after a prebake on a hotplate (Wenesco, Addison, IL, USA) at 160 °C for 4 min, the thickness of this resist film is 85 nm. The second one is AR-P 672.02. After the same prebake conditions, the total thickness of the two layers was 140 nm. E-beam lithography was performed with a stable electron-beam current of 200 pA and a dose of 300 µC/cm^2^. The development process was carried out in a solution of methyl isobutyl ketone and isopropyl alcohol in a ratio of 1:3. Isopropyl alcohol was used as a stop solution. Then, aluminum was thermally evaporated onto the samples, this process took place in the chamber at a pressure better than 8 × 10^−6^ Torr with a deposition rate of 1 A/s to obtain a 50 nm layer. Next, the substrates with the metal on top were immersed in acetone for the liftoff. As a result, the samples had the metal mask in the form of future pillars for reactive-ion etching. Etching occurred at a pressure of 5 mTorr, and, after, the aluminum was rinsed off in AZ 726 MIF Developer (Merck KGaA, Darmstadt, Germany). Further, substrates were cleaned with acetone and isopropyl alcohol in an ultrasonic bath again to remove residues from the developer. Finally, the layers of Cr, Ag, and Au, with respective thicknesses of 7, 40, and 15 nm, were successively evaporated onto the samples at pressures of more than 10^−7^ Torr at a rate of 1 A/s. This final step was performed just before taking the measurements.

### 3.3. Viruses

The following viruses were provided by the Chumakov Federal Scientific Center for Research and Development of Immune and Biological Products of the Russian Academy of Sciences: (1) influenza A virus (IvA), A/chicken/Rostock/45/1934 strain, H7N1 subtype with an activity of 256 HAU/mL; (2) influenza B virus (IvB), B/Victoria/2/1987 with an activity of 128 HAU/mL; Newcastle disease virus (NDV), vaccine strain with an activity of 128 HAU/mL. The virus stock was propagated in an allantoic cavity of 10-day-old embryonated specific pathogen-free chicken eggs. The eggs were incubated at +37 °C, cooled to +4 °C over the 48 h post-infection period, and harvested 16 h later. The study scheme was approved by the Ethics Committee of the Chumakov Institute of Poliomyelitis and Viral Encephalitides, Moscow, Russia (Approval #4 from 2 December 2014). The virus was deactivated by 0.05% (*v*/*v*) glutaric aldehyde, preserved by adding 0.03% (*w*/*v*) NaN_3_, and stored at +4°C.

The SARS-CoV-2 virus delta variant (B.1.617.2 strain) was provided by N. F. Gamaleya Federal Research Center for Epidemiology and Microbiology. The virus was grown on a Vero E6 (ATCC CRL-1586) cell line in a complete Dulbecco’s modified eagle’s medium with 10% fetal bovine serum, L-glutamine, and penicillin/streptomycin solution (100 IU/mL; 100 µg/mL). The virus titer was found to be 1.0 × 10^3^ TCID_50_/mL, determined as TCID_50_ by the endpoint dilution assay. The RNA detection of SARS-CoV-2 was carried out by means of a real-time PCR device, Rotor Gene 6000 (Corbett Research, Mortlake, Australia), using a set of reagents ‘SARS-CoV-2/SARS-CoV’ (DNA Technology, Moscow, Russia). The number of virions was estimated as the number of genome copies (1 × 10^8^ VP/mL). All the experiments with the live SARS-CoV-2 were conducted according to the approved standard operating procedures of the NRCEM biosafety, level-3 facility. Viruses were inactivated by 1% paraformaldehyde.

The HEp-2 and HeLa cells were cultured in complete RPMI and DMEM media, respectively. The A2 strain of the human respiratory syncytial virus (RSV), the type 3, type 5, and type 7 of the human adenoviruses (ADV 3, ADV 5, and ADV 7, respectively) were cultured on HEp-2 and HeLa cells, accordingly, in the medium containing 2% FCS. In brief, the day before infecting the HEp-2 and HeLa cells, they were seeded in 25 cm^2^ flasks and left to grow to confluence overnight at +37 °C and 5% CO_2_. The next day, the medium was removed from the flask, and the monolayer was gently washed with 5 mL of the serum-free medium. The cells were then infected with RSV or ADV. The flasks were left at +37 °C for 2 h, being gently shaken. After that, 10 mL of the medium containing 2% FCS was added to each flask, and they were left for another 36–48 h at +37 °C and 5% CO_2_. For the RSV-infected culture, the cells were harvested with a cell scraper, detaching 50% of the cells and revealing the formation of syncytia. Then, the cells were transferred into a 50 mL falcon tube to be sonicated for 3 min at +4 °C. For the ADV infected culture, the cells were also harvested using a cell scraper, detaching 50% of the cells, followed by two freeze–thaw cycles at -80°C. For both the RSV- and ADV-infected cultures, the cells were cleaned by centrifugation at 4000 RPM for 5 min at +4 °C. The supernatants containing the inactivated virus were then stored at +4 °C. In the cases of RSV and ADV, we used the viral stocks at 6.3 × 10^5^ and 2.9 × 10^6^ TCID_50_/mL, respectively. The viruses were deactivated by 0.05% (*v*/*v*) glutaric aldehyde.

### 3.4. Determination of Viral Particles in the Sample

Nanoparticle tracking analysis (NTA) was conducted using a ZetaView^®^ PMX420-QUATT instrument (Particle Metrix GmbH, Inning am Ammersee, Germany), while the data were analyzed by ZetaView NTA software. The operating instructions of the manufacturer were followed before calibrating the instrument with a known concentration of 100 nm polystyrene nanoparticles (Applied Microspheres B.V., Leusden, Netherlands). The standards were suspended in particle-free water, whereas the investigated samples were diluted 1:100 with PBS. Particles were counted and size-distributed at 10 cycles of 11 frames per cycle under a sensitivity of 65 and a shutter value of 100. The results are shown in Table 5.

### 3.5. Assembly of Aptamers

The assembly of the aptamer structure was conducted according to the following algorithm. First, the aptamers were prepared in 1 µM concentrations in the PBS buffer at pH 7.4, with 8 mM of Na_2_HPO_4_, 1.5 mM of KH_2_PO_4_, 140 mM of NaCl, and 10 mM of KCl. The solutions were heated to 95 °C, maintained at that temperature for 5 min, and then cooled to room temperature. Finally, the solutions were diluted with PBS to produce 200 nM solutions of the aptamer. All the solutions were made with ultrapure water provided by Millipore (Merck Millipore, St. Louis, MO, USA).

### 3.6. Biolayer Interferometry

Biolayer interferometry assays were performed using BLItz (ForteBio, Fremont, CA, USA). Streptavidin biosensors (ForteBio, Fremont, CA, USA) were hydrated in buffer A prior to the experiment. The protocol for the estimation of aptamer specificity was as follows: (1) initial baseline in PBS for 30 s; (2) loading of the aptamer from a 1 µM solution in PBS for 120 s; (3) the second baseline in PBS with 0.01% (*v*/*v*) tween-20, 0.1 mg/mL bovine serum albumin for 30 s; (4) association step in different dilutions of the viruses in PBS with 0.01% (*v*/*v*) tween-20, 0.1 mg/mL bovine serum albumin for 200 s; (5) dissociation step in PBS with 0.01% (*v*/*v*) tween-20, 0.1 mg/mL bovine serum albumin for 300 s.

The measurements were carried out in black tubes (Sigma-Aldrich, New York, NY, USA) with at least 220 µL of a respective solution in two-three repeats. The curves were approximated exponentially using a 1:1 binding model using exponential curves according to the guidelines for surface plasmon resonance [41]. The representation of binding curves for pairs of the aptamer-virus and several representative nonspecific pairs are provided in the text.

### 3.7. Aptasensor Maintenance

The spots of SERS substrate were incubated in 20 µL of 200 nM solution of the aptamer (SH-RHA0386-Cy3, SH-RBD1C-Cy3, SH-H8-TAMRA or SH-ADV-20-Cy3) for 15 min. The aptamer solution was removed using a piece of filter paper. The substrate was rinsed with 20 µL of ultrapure water and the drop was dried off with a piece of filter paper. The aptamer-modified substrates were prepared prior to the experiment.

### 3.8. SERS Measurements

The aptamer-modified SERS substrates were incubated with 1 mL of the mixtures of the viruses prepared in 10^3^–10^5^ dilution for 10 min. The culture medium from the noninfected Vero E6 cells diluted 10^3^ times with PBS was used for a virus dilution to minimize the effect of the off-target interactions. The samples were washed with 1 mL of water for 5 min. Then the wet substrates were covered with a cover glass and placed onto the SERS microscope table.

The SERS spectra were acquired with an Olympus BX51 optical scanning microscope (Olympus Corporation, Japan) based on the RamanLife RL532 spectrometer (TeraSense Group, Inc., San Jose, CA, USA) with 532 nm laser wavelength and 5 mW output power. The spectrometer provided a spectral resolution of 4–6 cm^−1^ over the spectral range of 160–4000 cm^−1^, at the laser spot of 10 µm in diameter. The map of the SERS signal distribution, 90 µm × 90 µm in size, was recorded over the entire area of the sample employing a 10× objective with the step of 15 µm in the XY-scanning mode. As a result, we obtained the maps of the intensity distribution for the Raman line (1586 cm^−1^) and the luminescence (1520 cm^−1^) of Cy3 or the Raman line (1645 cm^−1^) and the luminescence (1300 cm^−1^) of TAMRA. Then, we calculated the average values of the data as well as the measurement errors associated with each value.

### 3.9. Study of Surface Topology

SEM images were obtained using the scanning electron microscope Supra 50VP (Zeiss, Germany). The electron optical GEMINI column provided excellent beam brightness with an ultrahigh resolution of 1 nm at the accelerating voltage of 20 kV. Substrate surfaces and profiles were scanned at the accelerating voltage of 10 kV, with an aperture size of 30 µm, at the work distance of 8 mm, and at the chamber pressure of 9 × 10^−4^ Pa.

## Figures and Tables

**Table 1:** Dissociation constants of complexes aptamer virus. The constants of complexes of aptamers with their targets are shown in the grey filling.

Aptamer	Influenza A Virus	ADV 5	RSV	SARS-CoV-2
RHA0385	(2.1 ± 0.3) × 10^−14^	Low binding	Low binding	Low binding
ADV-20	(2.6 ± 0.2) × 10^−12^	(1.1 ± 0.2) × 10^−14^	(3.8 ± 0.5) × 10^−13^	(5 ± 2) × 10^−11^
H8	(3.5 ± 1.1) × 10^−12^	Low binding	(3.1 ± 0.8) × 10^−14^	Low binding
RBD-1C	Low binding	Low binding	Low binding	(7.1 ± 0.6) × 10^−14^

**Table 2:** The results of decoding the mixtures of adenovirus, influenza A, virus, SARS-CoV-2, and RSV A2 using a multiplex lithographic SERS aptasensor. The SERS to SEL ratio was used as the main parameter for the evaluation. The interpretation is shown in column ‘Result’. Neg. means a negative answer, i.e., the absence of the target virus; and pos. means a positive answer, i.e., detection of the target virus.

# of the Sensor	Spot	Detectable Virus in the Spot	Virus Content, VP/mL	Analytical Signal from Labeled Aptamers			
				SERS, a.u.	SEL, a.u.	SERS/SEL	Result
1	a	Adenovirus type 5	0	11,800 ± 500	29,100 ± 700	0.41 ± 0.01	≤0.44 → neg.
	b	Influenza A H7N1	0	5300 ± 200	18,800 ± 600	0.28 ± 0.01	≥0.25 → neg.
	c	SARS-CoV-2	1 × 10^5^	2800 ± 100	9200 ± 300	0.31 ± 0.01	<0.32 → pos.
	d	RSV A2	3 × 10^6^	2600 ± 100	12,200 ± 300	0.21 ± 0.01	<0.31 → pos.
2	a	Adenovirus type 5	7 × 10^5^	10,000 ± 400	17,900 ± 700	0.56 ± 0.01	>0.44 → pos.
	b	Influenza A H7N1	6 × 10^6^	3900 ± 100	19,500 ± 300	0.20 ± 0.01	<0.25 → pos.
	c	SARS-CoV-2	0	2700 ± 100	8200 ± 200	0.33 ± 0.01	≥0.32 → neg.
	d	RSV A2	0	2500 ± 100	7800 ± 500	0.32 ± 0.01	≥0.31 → neg.
3	a	Adenovirus type 5	0	10,000 ± 400	23,300 ± 800	0.43 ± 0.01	≤0.44 → neg.
	b	Influenza A H7N1	6 × 10^6^	5100 ± 100	2300 ± 300	0.22 ± 0.01	<0.25 → pos.
	c	SARS-CoV-2	1 × 10^4^	2900 ± 200	12,500 ± 300	0.24 ± 0.01	<0.32 → pos.
	d	RSV A2	3 × 10^5^	2600 ± 100	8500 ± 200	0.30 ± 0.01	<0.31 → pos.
4	a	Adenovirus type 5	7 × 10^4^	8800 ± 400	15,800 ± 500	0.56 ± 0.01	>0.44 → pos.
	b	Influenza A H7N1	6 × 10^5^	3500 ± 100	15,200 ± 100	0.23 ± 0.01	<0.25 → pos.
	c	SARS-CoV-2	1 × 10^3^	3400 ± 200	10,800 ± 200	0.31 ± 0.01	<0.32 → pos.
	d	RSV A2	0	2300 ± 100	6800 ± 200	0.33 ± 0.01	≥0.31 → neg.
5	a	Adenovirus type 5	0	8200 ± 300	26,200 ± 900	0.31 ± 0.01	≤0.44 → neg.
	b	Influenza A H7N1	6 × 10^6^	3700 ± 100	18,700 ± 100	0.20 ± 0.01	<0.25 → pos.
	c	SARS-CoV-2	1 × 10^5^	2900 ± 100	11,300 ± 500	0.26 ± 0.01	<0.32 → pos.
	d	RSV A2	0	2400 ± 100	7000 ± 200	0.33 ± 0.01	≥0.31 → neg.
6	a	Adenovirus type 5	7 × 10^5^	11,700 ± 300	21,000 ± 900	0.56 ± 0.01	>0.44 → pos.
	b	Influenza A H7N1	0	4900 ± 100	16,700 ± 100	0.29 ± 0.01	≥0.25 → neg.
	c	SARS-CoV-2	0	2800 ± 200	8200 ± 300	0.34 ± 0.01	≥0.32 → neg.
	d	RSV A2	3 × 10^6^	2400 ± 100	8300 ± 100	0.29 ± 0.01	<0.31 → pos.

**Table 3:** Comparison of analytical performance in the detection of viral particles using aptamer-based SERS sensors, antibody-based SERS sensors, DNA probe-based SERS, SERS sensors without recognition elements, and conventional diagnostic assays. N/A—not applicable, RT PCR—reverse transcription polymerase chain reaction, RT LAMP—reverse transcription loop-mediated isothermal amplification. The recalculations of viral concentrations were performed using the ratios from [21,22,23,24]; the molecular weight of the viruses was approximated as 10^7^ Da. N/A—not applicable.

Analytical Technique	Virus Type	Analytical Performance					Ref.
		Limit of Detection		Quantification	Time of Analysis	Multiplex Analysis	
		Observed	Recalculated				
SERS without recognizing elements	Rheovirus, rinovirus, influenza A, parainfluenza	10^2^ EID_50_/mL	10^4^ VP/mL	No	15 min	Not reported	[25]
	Respiratory syncytial virus	100 pfu/mL	3 × 10^5^ VP/mL	Yes	1 h	Not reported	[26]
	Circovirus, parvovirus, pseudorabies	1 × 10^7^ VP/mL	1 × 10^7^ VP/mL	No	15 min	Not reported	[27]
Aptamer-based SERS	Influenza A	2.2 × 10^−5^ HAU/mL	10^3^ VP/mL	No	15 min	Not reported	[11]
		10 VP/mL	10 VP/mL	Yes	15 min	Not reported	[28]
	Influenza A/ SARS-CoV-2	1.06 HAU/mL 0.95 pfu/mL	5 × 10^7^ VP/mL 7 × 10^5^ VP/mL	Yes	15 min	Yes	[29]
	SARS-CoV-2	100 copies/mL	100 VP/mL	Yes	15 min	Not reported	[8]
	Influenza A/ SARS-CoV-2/ RSV/ Adenovirus	600 VP/mL 100 VP/mL 3 × 10^3^ VP/mL 70 VP/mL	600 VP/mL 100 VP/mL 3 × 10^3^ VP/mL 70 VP/mL	No	17 min	Yes	This work
Antibody-based SERS	Influenza A	4 × 10^3^ TCID_50_/mL	4 × 10^5^ VP/mL	Yes	3.5 h	Not reported	[30]
		30 ng/mL	1.8 × 10^8^ VP/mL	Yes	2 h	Not reported	[31]
	Influenza A/ Adenovirus	50 pfu/mL 10 pfu/mL	3 × 10^7^ VP/mL 500 VP/mL	Yes	30 min	Yes	[4]
	Ebola/Lassa viruses	10^5^ pfu/mL	10^6^ VP/mL	Yes	30 min	Yes	[5]
	Influenza A/ SARS-CoV-2/ RSV	85 copies/mL 8 pg/mL 8 pg/mL	85 VP/mL 5 × 10^4^ VP/mL 5 × 10^4^ VP/mL	Yes	30 min	Yes	[7]
	Human immunodeficiency virus	35 pg/mL	2 × 10^5^ VP/mL	Yes	12 h	Not reported	[32]
DNA probe-based SERS	Influenza A and B/parainfluenza/RSV/parainfluenza/adenovirus	30–41 fM	2 × 10^7^ VP/mL	Yes	10 min	Yes	[6]
RT PCR	Influenza A	3 × 10^2^–1.2 × 10^3^ VP/mL	3 × 10^2^–1.2 × 10^3^ VP/mL	Yes	2–3 h	Yes	[33,34]
	SARS-CoV-2	10^2^–1.2 × 10^3^ VP/mL	10^2^–1.2 × 10^3^ VP/mL	Yes	2–3 h		[34,35,36]
RT LAMP	Influenza A	10^4^ VP/mL	10^4^ VP/mL	Yes	1 h	Yes	[37]
	SARS-CoV-2	2 × 10^4^ VP/mL	2 × 10^4^ VP/mL	Yes	1 h		[38]
Antibody-based test strip	Influenza A	20 TCID_50_/mL	1 × 10^6^ VP/mL	No	10–15 min	Yes	[39]
	SARS-CoV-2	7.6 × 10^3^ TCID_50_/mL	5 × 10^8^ VP/mL	No	15 min		[40]

**Table 4:** Sequences of DNA oligonucleotides used in the study. The known aptamer targets are shown.

Code	Sequence (5→3)	Target
Biotin-RHA0386	(Biotin)-TTGGGGTTATTTTGGGAGGGCGGGGGTT	Hemagglutinin of influenza A
SH-RHA0386-Cy3	(SH-(CH_2_)_6_)-TTGGGGTTATTTTGGGAGGGCGGGGGTT-(Cy3)	Hemagglutinin of influenza A
Biotin-RBD-1C	(Biotin)-CAGCACCGACCTTGTGCTTTGGGAG TGCTGGTCCAAGGGCGTTAATGGACA	S protein of SARS-CoV-2
SH-RBD-1C-Cy3	(SH-(CH_2_)_6_)-CAGCACCGACCTTGTGCTTTGGGAG TGCTGGTCCAAGGGCGTTAATGGACA-(Cy3)	S protein of SARS-CoV-2
Biotin-H8	(Biotin)-AGTGCGGTGAGCCGTCGGACATACAAATAC	RSV A2 virions
SH-H8-TAMRA	(SH-(CH_2_)_6_)-AGTGCGGTGAGCCGTCGGACATACAA ATAC-(TAMRA)	RSV A2 virions
Biotin-ADV-20	(Biotin)-GTGCCAGCTATGCCATTGGCGGGTCGTCC AATTCGAGAGGTCCCCTAGCGTCTATCTCTGCTGC	Adenovirus type 2
SH-ADV-20-Cy3	(SH-(CH_2_)_6_)-GTGCCAGCTATGCCATTGGCGGGTCGT CCAATTCGAGAGGTCCCCTAGCGTCTATCTCTGCTGC-(Cy3)	Adenovirus type 2

**Table 5:** Number of viral particles in biological samples according to nanoparticle tracking analysis or reverse transcription polymerase chain reaction (marked with *) assays.

Virus	Number of Virions per mL
Influenza A virus	6.1 × 10^9^
SARS-CoV-2	1.0 × 10^8^ *
Respiratory syncytial virus	3.1 × 10^9^
Adenovirus 5	7.4 × 10^8^
Influenza B virus	6.0 × 10^9^
Newcastle disease virus	3.6 × 10^10^

## Data Availability

The data that support the findings of this study are available from the corresponding authors upon reasonable request.
